# Torsional Resonance Vibrations of Uniform Bars of Square Cross Section

**DOI:** 10.6028/jres.065A.020

**Published:** 1961-06-01

**Authors:** Wayne E. Tefft, Sam Spinner

## Abstract

Relations by which the shear modulus may be computed from the fundamental and overtones of the torsional resonance frequencies of square bars have been established empirically.

The results are analyzed in terms of a proportionality factor, *R*, defined by the equation *G* = (2*l f_n_*/*n*)^2^*ρR. R* is found to increase with increasing cross section to length ratio. Also, the overtones are less than integral multiples of the fundamental by an amount which increases with increasing cross section to length ratio.

## 1. Introduction

### 1.1. General Background

This is the third in a series of papers [1,2]^1^ dealing with the relations between various mechanical resonance frequencies of uniform bars and their elastic moduli. The general method of approach has been described in the previous papers. Essentially this approach consists in determining the mechanical resonance frequencies of a series of specimens, all having the same intrinsic elastic moduli and density, but having dimensions which differ from each other in a consistent manner. Then, relations are developed from which the elastic moduli can be computed from the associated resonance frequencies in terms of properly selected dimensional and elastic parameters. These empirically established relations are compared with corresponding theoretical ones (based on the classical theory of elasticity) whenever feasible. The theoretical relations serve as a guide in the selection of these foregoing parameters. Also, it is only because the experimental methods for determining these resonance frequencies have been developed to a high degree of accuracy (see sec. 2.2) that it has become possible to develop the empirical relations with comparable accuracy, and to check the classical theory more carefully than has been possible heretofore.

Since the uniformity of specimens with respect to elastic modulus and density is a necessary condition for the entire development, considerable care must be taken in the selection of specimens to realize this condition experimentally. If, in the course of the investigation, an opportunity presents itself for checking this uniformity independently, it is clearly of the utmost value, since, as just indicated, failure of the specimens to conform to this condition would render the entire subsequent analysis invalid. Such an opportunity occurred in a previous study (specimens of set II in [2]) and again in this study, as will be shown.

Generally, steel specimens have been chosen because this material possesses certain desirable properties in fulfilling the purposes of these investigations. These include,

1. Steel can be machined to high dimensional accuracy fairly easily.
2. The material is inexpensive. (For these reasons steel is chosen over tungsten, for instance.)
3. It is dimensionally staple.
4. It has been found that if reasonable care is taken, the fundamental condition of maintaining the uniformity among the different specimens with respect to intrinsic modulus and density can be achieved to the high accuracy necessary for the subsequent analysis.
5. Also, if sufficient care is taken, steel can be selected which satisfactorily fulfills the assumptions on which the empirical and corresponding theoretical equations are based, namely, that the material be homogeneous and isotropic. Even though the individual grains of which the steel is composed are anisotropic, and chemically, the material is not “pure”, nevertheless, the orientation and distribution of the grains is random. On the macroscopic scale of the experiments and analysis, this material may safely be considered to be isotropic and homogeneous, and equations based on this condition are valid.
6. Steel gives excellent elastic responses under the experimental conditions used, so that resonance frequencies of up to about 50 kc/s can be realized. This applies for higher overtones of longer specimens or lower overtones or the fundamental of the shorter specimens. Consequently, a fairly wide range of experimental data can be analyzed. This is of considerable importance, because it is usually only at higher frequencies that departures from the theory are large enough to be observed.

It is emphasized, however, that although steel specimens have been used almost entirely in these investigations (and will be used in this one), the results are not peculiar to steel, but should apply to any elastic, homogeneous, isotropic material. The only qualification to this statement is that for Young’s modulus and its related modes of vibration, flexural and longitudinal, Poisson’s ratio must also be considered. This complication presumably does not arise in the determination of shear modulus from measurements in the first torsional mode of any order.

### 1.2. Particular Problem of This Study

It is recalled from the previous paper [1] on torsion that the first overtone of rectangular specimens was found to deviate from the exact double of the fundamental, and that the amount of deviation varied with the width to depth ratio of the cross section. Even for a specimen of square cross section the data revealed that the first overtone was slightly lower than the exact double of the fundamental. However, the data of that paper [1] were limited to one length of specimen (about 6 in.) and to only the first overtone. The purpose of the present paper is to consider this problem more fully by investigating specimens of square cross section of different lengths and at higher overtones.

To give a fuller picture, the fundamental and higher overtones of torsional resonance of some cylindrical specimens were also investigated.

## 2. Experiment

### 2.1. Specimens

All the specimens listed were cut from a single bar of steel, designated as SAE 1010. It was the same bar mentioned in footnote 4 in [2]. Its composition, as determined by spectroscopic analysis was, carbon 0.10 percent, manganese 0.30 percent, phosphorus 0.011 percent, sulfur 0.022 percent, and the remainder, iron. The original bar was about 2 ft long and about 1½ in. square in cross section. The steel was specially heat treated to be as homogeneous as possible and to have a minimum of preferred crystal orientation. As further precaution to insure uniformity, the original specimens were cut from the center of the stock, since preferred crystal orientation, when it does occur, is usually most pronounced at the periphery.

First, two cylindrical and one square specimen were machined from the original stock. After the fundamental and as many overtones as possible were determined for these specimens, the square one was subdivided in length and the torsional resonances were similarly determined for the two new shortened specimens thus formed. The larger of these two specimens was again subdivided and the resonances once more determined. The subdivisions were so performed that the resulting specimens were all of different lengths.

In this manner, a large number of specimens (and, therefore of experimental points) was made available from the original bar stock. The subscripts in the specimen designations indicate the history of the subdivision process. Thus A12, indicates that the specimen was cut from A1, A1 was cut from A, which in turn was cut from the original bar.

The dimensions of all the specimens were true to ±0.0003 cm. This was a higher order of accuracy than that achieved in the previous investigations. The accuracy of the dimensions and the intrinsic uniformity of the specimens was such that the standard deviation in the density of four randomly selected specimens, calculated from the mass and volume (obtained from the dimensions) was less than 0.0002 g/cm^3^, the average density being 7.8541/g/cm^3^.

### 2.2. Resonance Frequencies

The torsional resonance frequencies were obtained in the manner previously described [1]. In addition to the precautions previously taken to insure accuracy, sufficient time was allowed to elapse for the specimens to arrive at equilibrium with the ambient temperature which was controlled at 25.0±0.5°C. This often took from 2 to 3 hr since it was found that significant frequency variations could be observed during this time interval. The specimen was considered to have come to equilibrium when successive measurements at one-half hour intervals showed no change in the measured resonance frequencies (i.e., one in the last significant figure). When this additional (temperature) precaution was taken, a conservative figure for the accuracy of the resonance frequency determinations was about 1 part in 10,000. This figure is about 2½ times better than that given previously [1,2].

Table 1 lists the dimensions, and torsional resonance frequencies, both fundamental and overtones, of the specimens used in this investigation.

## 3. Results and Discussion

### 3.1. General Theory

All rods, whether cylindrical, square or rectangular in cross section conform to the following equation relating the shear modulus, *G*, to the density, *ρ*, the first (nondispersive)-mode torsional resonance frequency, *f*, and the length, *l*, of the specimens,

$$G={\left(2lf_{n}/n\right)}^{2}\rho R$$ (1)

*n* signifies the overtone of the resonance frequency; for the fundamental, *n* = 1, first overtone, *n* = 2, etc. *R* is a proportionality factor which depends upon the shape of the specimen and *n.*

### 3.2. Cylindrical Specimens

For cylindrical specimens, the theory states that *R* = 1 exactly for all length to cross section ratios and for all overtones. Also torsional waves in cylinders conform to the following two equations (which are exactly analogous to the ones for longitudinal waves in cylinders),

$$n\lambda =2l\;\text{and}\;\upsilon_{t}=\frac{2lf_{n}}{n}$$ (2)

where λ = wavelength and *υ_t_* = velocity, of torsional waves.

In contrast with longitudinal waves in cylinders, however, the velocity of torsional waves is not reduced as the length of the specimen is reduced with respect to the cross section. For all lengths, then,

$$G={\upsilon_{t}}^{2}\rho .$$ (3)

The constancy of *R* was checked from the experimental data by substituting the appropriate values for the two cylindrical specimens, in eq (1) and assuming *G*/*ρ* to be the same for both specimens. *R* was found to be constant with a coefficient of variation of 1.2×10^−7^ percent. Since these two specimens were not long enough to provide enough overtones for an extended range of data, the constancy of *R* was further checked experimentally by using another pair of specimens, about 12 in. and 8 in. long, both about 1 in. in diameter from an earlier source (specimens I–19 and I–16 from [2]). For the longer of these specimens, torsional resonances up to the fourth, and for the shorter specimen, up to the third overtone could be detected; and *R* was similarly computed from these 9 resonances. Again, *R* was found to be constant with a coefficient of variation of 1.1×10^−7^ percent. Furthermore, *R* showed no tendency to drift systematically for either pair of specimens.

The equations for torsional waves in cylinders are not only exact hut so clear cut and simple (requiring no qualifying correction factors) that, had the experimental results not been in accord with the theory, one would be more inclined to suspect the data than the theory. Consequently, the excellent agreement found in this respect is reassuring evidence of the reliability of the experimental data.

*G*/*ρ* for the specimens may now be obtained by substituting the 3 sets of values for the two cylindrical specimens in eqs (2) and (3). The average of these 3 values so obtained is,

$$\frac{G}{\rho }={\upsilon_{t}}^{2}=(104.117\pm .008)\times 10^{9}{\left(\frac{\text{cm}}{\sec}\right)}^{2}.$$ (4)

Since *ρ* is known from section 2.1, *G* may also be computed. It turns out to be 817.7×10^9^ dynes/cm^2^. However, this information is not necessary for the further development.

### 3.3. Square Bars

For torsional waves in bars of square cross section, unfortunately, the situation is not so simple as in cylinders. (Not only is the theory more complex requiring uncertain approximations, but the experimental results obtained here do not agree too well with the theory.)

Timoshenko [3] has derived exact expressions for the stress, strain and dimensional relations of square bars in static torsion. Timoshenko’s equations have been solved for a shape factor, *k*_1_, which when substituted into Pickett’s [4] equation for *R*, (*R* = *I_p_*/*k*_1_ where *I_p_* is the polar moment of inertia of the cross-sectional area) leads to a value of *R* = 1.18559, which is accurate to the number of places given. This particular value of *R* is henceforth designated as *R*_0_. For the dynamic case (i.e., a square bar vibrating in torsion) *R*_0_ may be safely used in eq (1) as long as the cross section of the specimen is small in comparison with the wavelength. For shorter specimens in torsional resonance, however, as the cross section becomes a significant part of the wavelength, the strain pattern departs sufficiently from the static case to require modification (increase) in the value of *R*_0_.

Davies [5] has considered theoretically this problem of change in *R* for shorter square specimens along with the possible departure of the overtones from integral multiples of the fundamental. His concluding equation, which involves a number of approximations, may be expressed, in the notation of this paper, as follows:

$$R/R_{0}=1+.00851{\left(n\frac{t}{l}\right)}^{2}$$ (5)

where *t* = the length of a cross sectional edge.

It is clear from Davies’ equation and eq (1), that as *t*/*l* increases *R*/*R*_0_ will increase and the overtones will decrease by greater amounts from integral multiples of the fundamental. These results are in qualitative agreement with the experimental data. However if one attempts to fit the experimental results to Davies’ equation, one finds significant quantitative disagreement, especially at higher values of *t*/*l* and at higher overtones.

**Figure 1:**
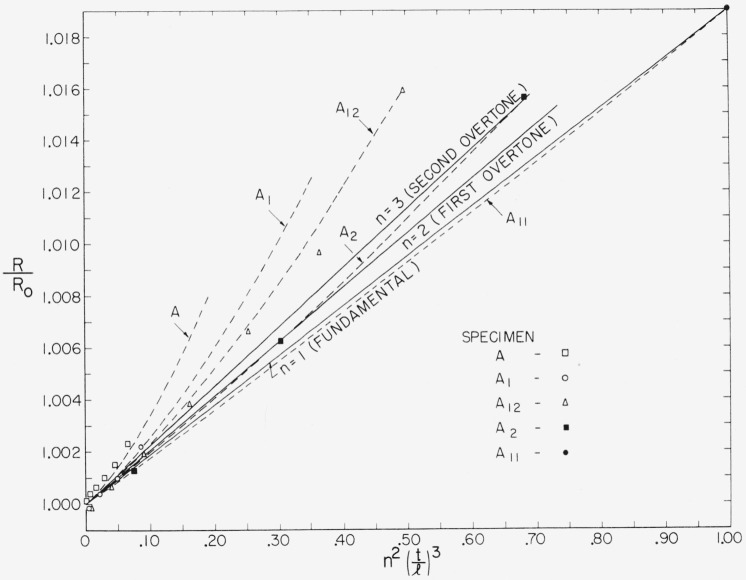
Plot of correction factor *R/R_o_* for computing the shear modulus of square bars from their torsional resonance frequencies. *R*/*R_o_* is a function of *n*, *t*, and *l* where; *n* is the order of the overtone, *t* is length of the cross sectional edge, and *l* is the length of the specimen. The dashed lines are for constant *t*/*l.* The solid lines are for constant *n.* All the lines are actually drawn by solving the equation, *R*/*R_o_* = 1+*n*^2^(*t*/*l*)^3^ (0.01746+0.00148*n*+0.00009*n*^2^) for selected values of *n* or *t*/*l. R_o_* = 1.18559.

After considerable manipulation, it was found that a satisfactory fit could be obtained if *R*, or preferably *R*/*R*_0_, was not assumed to be a function of (*nt*/*l*)^2^ as Davies does, nor any other function of (*nt*/*l*), but rather a function of *n* and *t*/*l* separated. Figure 1 is a plot of *R*/*R*_0_ as a function of *n*^2^(*t*/*l*)^3^.

For any given value of *n*, *R*/*R*_0_ varies very nearly as (*t*/*l*)^3^. Therefore the solid lines in the figure for *n* = 1, *n* = 2, and *n* = 3 (which plot *R*/*R*_0_ as functions of (*t*/*l*)^3^) are very nearly straight lines. The 5 dashed lines represent the variations in *R*/*R*_0_ for given values of *t*/*l* (represented by the values for the 5 square specimens) as a function of their overtones. It is seen from the figure that for constant *t*/*l*, *R*/*R*_0_ is not a linear function of *n*^2^, but requires further modification. The equation finally arrived at, for most accurately representing all the data, was of the form,

$$R/R_{0}=A+n^{2}{\left(t/l\right)}^{3}(B+Cn+Dn^{2}).$$ (6)

The constant, *A*, in this equation would equal one, if the two basic assumptions made in arriving at the equation are correct. These are (1) that it is legitimate to substitute *R*_0_, solved from the static case, into the dynamic one for a long specimen, i.e., that *R*/*R*_0_→1 as *t*/*l*→0 and (2) that the numerical value of *G*/*ρ* given in eq (4) and contained implicitly in R is correct, for the square bars as well as for the cylinders.

In order for *A* to equal one, both of these assumptions must be correct, except in the unlikely possibility that both are incorrect in such a manner as to cancel each other. Also the second of these assumptions is recognized to be the one mentioned earlier as the basic condition for the entire analysis. Therefore if *A* = 1 is not assumed but is solved for, along with the constants *B*, *C*, and *D* from the available data, then the degree to which *A* approaches one, will be a critical indication of the correctness of these assumptions. This agreement of *A* with one is also the independent check mentioned earlier (sec. 1.1) on the uniformity of the specimens with respect to modulus and density (actually *G*/*ρ*).

The constants, *A*, *B*, *C*, and *D* were determined by a least squares routine on an automatic computer. For this purpose, the data from the same specimens, as plotted in figure 1, 21 measurements on 5 specimens were used.

The values so obtained for these constants are given below,

ConstantValueStandard error
*A*1.000100.00006*B*0.01745  .00045*C*  .00148  .00036*D*  .00009  .00005
Standard deviation for *R*/*R*_0_ = 0.00022.

The value for *A* is seen to depart only insignificantly from one. If *A* = 1 were used with the same standard error actually obtained for *A*, then *G*/*ρ* would come out to be 104.107±0.006. Had this value turned out to be significantly different from the one actually used, (*G*/*ρ* = 104.117±0.008) then a readjustment in the values for *B*, *C*, and *D* would be necessary. However, the difference between the two values for *G*/*ρ*, the one actually used, and the one resulting from assuming *A* = 1, is not statistically significant, being not greater than (actually equal to) the standard error of the difference which is

$$\sqrt{{\left(0.006\right)}^{2}+{\left(0.008\right)}^{2}.}$$

Therefore no such readjustment is necessary. It appears then that, except in the unlikely possibility mentioned above, the assumptions made in arriving at eq (6) are valid. Also, the standard deviation in *R*/*R*_0_, is well within the error to be expected on the basis of the accuracies given for the resonance frequencies and the dimensions of the specimens. The standard deviation of this variable is most critical since it is the test of whether the form of equation selected (eq (6)), accurately represents the data. Had the standard deviation of *R*/*R*_0_ been larger than that to be expected from the experimental error, it would have meant that the form of equation chosen was incorrect. The entire analytical expression corresponding to figure 1 may now be written as,

$$R/R_{0}=1+n^{2}{\left(t/l\right)}^{3}(0.01746+0.00148n+0.00009n^{2})$$ (7)

Actually the curves in the figure are not drawn through the experimental points but through values obtained by solving eq (7) for selected values of *n* and *t*/*l.* This gives a graphical indication of the degree of agreement between the equation and the experimental points.

## 4. Summary

Accurate relations have been developed from which the shear modulus may be computed from the torsional resonance frequencies of uniform bars of square cross section. These relations are in qualitative agreement with Davies’ corresponding theoretical formulation which recognizes that the proportionality factor *R* varies for different ratios of cross section to length and also for higher overtones. However the accurately determined empirical relations given here are shown to depart from Davies’ equation especially at these higher cross section to length ratios and higher overtones.

## Figures and Tables

**Table 1:** Dimensions and torsional resonance frequencies of steel bars of square cross section

Specimen^a^	*t*/*l*^b^	Resonance frequencies, c/s^c^						
		*f*_1_	*f*_2_	*f*_3_	*f*_4_	*f*_5_	*f*_6_	*f*_7_

A	0.12225	5182.0	10362.7	15542	20719	25892	31058	……….
A 1	.17497	7417.9	14832.0	22241	29637	……….	……….	
A 12	.21609	9161.5	18315.6	27456	36573	45653	54700	63621
A 2	.42339	17937	35785	53430	……….	……….	……….	……….
A 11	1.00000	41995	……….	……….	……….	……….	……….	……….
B	0.45039	28233	……….	……….	……….	……….	……….	……….
C	.27871	17471	34946	……….	……….	……….	……….	……….

a Specimens designated A are bars of square cross section, those designated *B* and *C* are cylinders of circular cross section.

b *l* = length of specimen, for square specimens *t* = cross-sectional edge = 3.4950 cm; for cylindrical specimens *t* = diameter = 2.5737 cm.

c *f*_1_ = fundamental torsional resonance frequency,

*f*_2_ = first overtone, etc.

## Footnotes

1 Figures in brackets indicate the literature references at the end of this paper.
